# Fibrosis to carcinogenesis: unveiling the causal dynamics between pulmonary fibrosis and lung cancer

**DOI:** 10.3389/fonc.2024.1452559

**Published:** 2024-08-16

**Authors:** Yiming Huang, Zhi Lin, Ting Huang, Heran Zhou

**Affiliations:** ^1^ Hangzhou Hospital of Traditional Chinese Medicine, Hangzhou TCM Hospital of Zhejiang Chinese Medical University, Hangzhou, Zhejiang, China; ^2^ Department of Gynecology, Hospital of Obstetrics and Gynecology, Fudan University, Shanghai, China; ^3^ Department of Oncology, Hangzhou TCM Hospital Affiliated to Zhejiang Chinese Medical University, Hangzhou, Zhejiang, China

**Keywords:** pulmonary fibrosis, lung cancer, Mendelian randomization, small cell lung cancer, non-small cell lung cancer

## Abstract

**Background:**

Previous clinical evidence has shown a correlation between pulmonary fibrosis (PF) and lung cancer (LC), but their causal relationship remains unknown.

**Methods:**

This study utilized a bidirectional two-sample Mendelian randomization (MR) approach to explore the causal relationship between PF and LC, including its subtypes. Genetic data were obtained from the IEU and FinnGen Genome-Wide Association Studies (GWAS). SNPs with genome-wide significance were selected, and analyses were conducted using Inverse-Variance Weighted (IVW), MR Egger, and Weighted Median methods. The IVW results for various subtypes of lung cancer and PF were used in a meta-analysis to investigate the overall causal effect between PF and lung cancer. Sensitivity analysis was used for both MR and meta-analysis to investigate the robustness of the results.

**Results:**

The bidirectional MR analysis showed no significant causal relationship between PF and overall, LC or its subtypes, except for SCLC, which had a significant positive association (OR = 1.29, 95% CI 1.07-1.57, p = 0.009). The meta-analysis results indicated no overall causal effect (OR = 1.067, 95% CI: 0.952-1.195, P = 0.265, I² = 57.3%). In the reverse MR analysis, NSCLC and LUSC showed significant associations with PF (OR = 1.12, 95% CI 1.01-1.23, p = 0.028 and OR = 1.04, 95% CI 1.01-1.08, p = 0.012, respectively), while the meta-analysis results indicated no significant causal effect (OR = 1.006, 95% CI: 0.973-1.040, P = 0.734, I² = 55.9%). Sensitivity analyses indicated no evidence of horizontal pleiotropy or significant heterogeneity.

**Conclusion:**

This study suggests a potential causal relationship between PF and SCLC, as well as between NSCLC and LUSC with PF. However, the overall causal relationship between PF and LC was not statistically significant, possibly due to individual variability and other influencing factors. Further research using data from diverse populations is needed to validate these findings.

## Introduction

With advancements in medical science, cancer mortality rates have gradually declined over recent decades. However, lung cancer (LC) continues to pose a significant threat to human life. In the United States, lung cancer remains the leading cause of cancer-related deaths, surpassing all other forms of cancer in mortality rates (1). LC is classified into small cell lung cancer (SCLC) and non-small cell lung cancer (NSCLC). NSCLC is further subdivided into lung adenocarcinoma (LUAD), lung squamous cell carcinoma (LUSC), and large cell carcinoma. LUAD is considered the most common subtype, while SCLC has the poorest prognosis (2–4). Although LC is classified into different subtypes based on pathology, they are all commonly associated with a clinical condition known as pulmonary fibrosis (PF) (5, 6).

In PF, healthy lung tissue is gradually replaced by fibrotic scar tissue, leading to stiffening and a decline in lung function (7). Idiopathic pulmonary fibrosis (IPF) is a specific type of pulmonary fibrosis (PF). A study conducted by JafariNezhad et al. found that the prevalence of lung cancer in IPF patients was as high as 13.54%. Among the histological subtypes, LUSC was the most predominant type associated with IPF, with a prevalence of 38.82%, followed by LUAD at 30.79% (8). Another study by Karampitsakos et al. found that during a ten-year follow-up of IPF patients, 26.6% of the surviving patients developed LC. Additionally, patients with both IPF and lung cancer often had worse clinical outcomes compared to those with only IPF (9).

It is obvious from multiple pieces of evidence that there is a correlation between PF and LC; however, the causal relationship between them remains unknown (10, 11). Mendelian randomization (MR), a research method capable of determining causality, has been employed to assess the causal relationship between IPF and lung cancer as well as its subtypes (12). Previous studies have identified a causal relationship only between IPF and lung adenocarcinoma. Various clinical correlations suggest that PF may be associated with multiple types of lung cancer. Given that IPF is a subtype of PF, and previous studies did not explore reverse causality, these studies are incomplete. Therefore, our research will employ bidirectional two-sample MR to explore the bidirectional causal relationship between PF and various lung cancer subtypes, thereby supplementing the conclusions of previous studies.

## Methods and materials

### Study design

**Figure 1:**
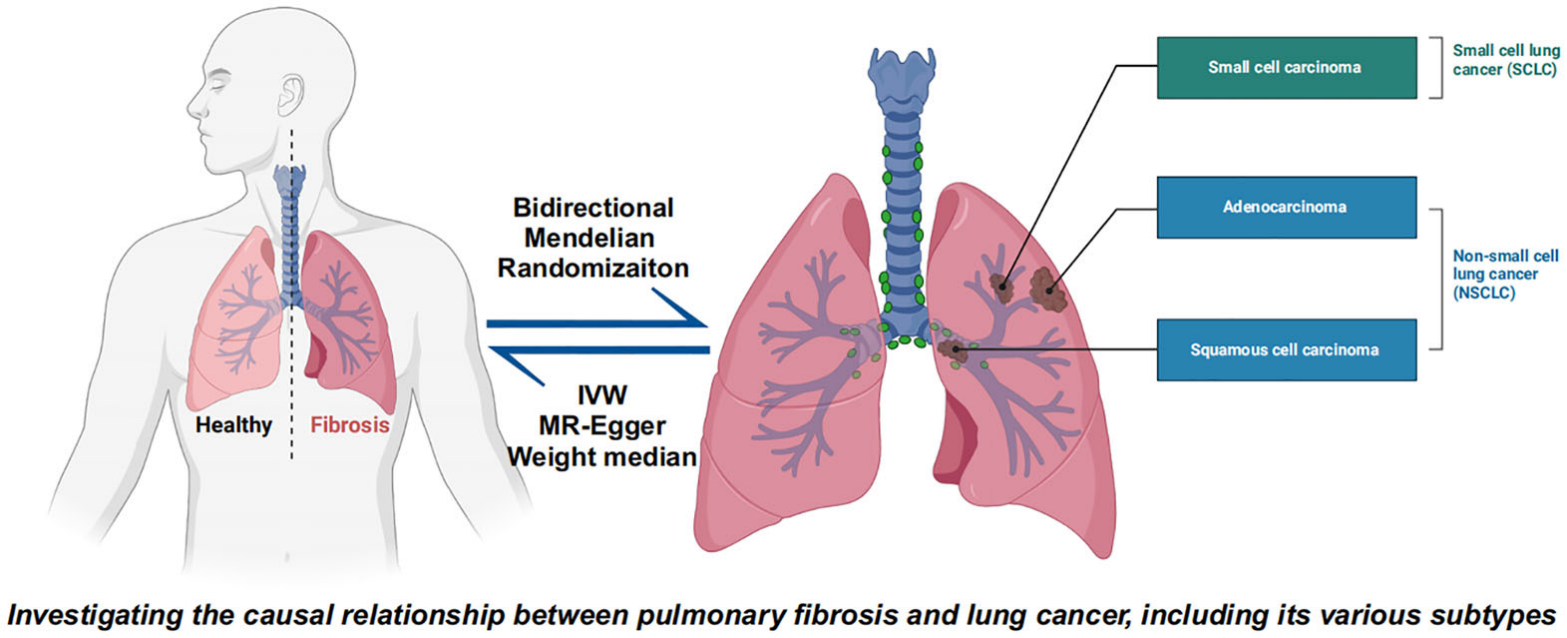
Bidirectional Mendelian randomization explores the causal relationship between pulmonary fibrosis and lung cancer and lung cancer subtypes including small cell lung cancer, non-small cell lung cancer, lung squamous cell carcinoma, and lung adenocarcinoma.

In this study, we employed a bidirectional two sample MR approach to investigate the potential bidirectional causal relationship between PF and LC as well as its subtypes. Initially, the SNPs involved in the study were selected based on fulfilling three key assumptions required for the analysis: 1) The genetic variants must be strongly associated with the exposure factor; 2) The SNPs should not be related to any confounding factors that may influence the risk factor-outcome association; 3) The SNPs must affect the outcome solely through the exposure of interest, and not via any alternative pathways. The study design was presented in **Figure 1**, the bidirectional causal relationships between PF and LC, including SCLC, NSCLC, LUSC and LUAD were investigated.

### Data source and genetic instruments selection

The data for pulmonary fibrosis, lung cancer and subtypes of lung cancer in this study were sourced from the IEU and FinnGen Genome-Wide Association Studies (GWAS). Detailed information about these GWAS studies is presented in **Table 1**. All datasets involved in this research are publicly available, and the original studies from which the data were derived have received ethical approval. This ensures the integrity and accessibility of the data used in our analysis.

**Table 1:** Sources of original GWAS data.

Phenotype	IEU-ID	population	sample size	SNP numbers	Database links
Pulmonary fibrosis	ebi-a-GCST90018908	European	469,126	24,195,349	https://gwas.mrcieu.ac.uk/datasets/ebi-a-GCST90018908/
Lung cancer	ieu-a-986	European	9,298	7,024,138	https://gwas.mrcieu.ac.uk/datasets/ieu-a-986/
Non-small cell lung cancer	finn-b-C3_LUNG_NONSMALL	European	\	16,380,466	https://gwas.mrcieu.ac.uk/datasets/finn-b-C3_LUNG_NONSMALL/
Small cell lung cancer	finn-b-C3_SCLC_EXALLC	European	\	16,380,303	https://gwas.mrcieu.ac.uk/datasets/finn-b-C3_SCLC_EXALLC/
Lung adenocarcinoma	finn-b-C3_NSCLC_ADENO_EXALLC	European	\	16,380,303	https://gwas.mrcieu.ac.uk/datasets/finn-b-C3_NSCLC_ADENO_EXALLC/
Lung squamous cell cancer	finn-b-C3_NSCLC_SQUAM_EXALLC	European	\	16,380,303	https://gwas.mrcieu.ac.uk/datasets/finn-b-C3_NSCLC_SQUAM_EXALLC/

To ensure the validity of the assumptions and the availability of usable data, as well as to guarantee an adequate number of usable SNPs for the study, this study selected Single Nucleotide Polymorphisms (SNPs) with genome-wide significance (p < 1 x 10^-5^) for both the forward and reverse analyses. A stringent linkage disequilibrium clustering algorithm with r^2^ < 0.001 and a 10000 kb window was employed to guarantee the independence of the instruments. For each SNP, the residual F-statistic was assessed, and only SNPs with an F-statistic greater than 20 were retained. This approach was crucial to ensure the robustness and reliability of the MR analysis.

### Statistical analysis

In the primary analysis of our study, we employed three mainstream Mendelian Randomization methods: Inverse-Variance Weighted (IVW), MR Egger, and Weighted Median. The IVW method, applied with a random effects model, was the primary analytical tool (13). Meanwhile, the final IVW results of different subtypes of lung cancer from the bidirectional analysis were used for a meta-analysis to assess the overall effect. Since NSCLC includes LUSC and LUAD among its subtypes, to avoid duplication in the meta-analysis, data on NSCLC were not included in the same meta-analysis model as data on LUSC and LUAD simultaneously. Heterogeneity was considered present if the I² value was greater than 50% or if the p-value of Cochran’s Q test is less than 0.05. In such cases, the random-effect model should be selected as the relatively reliable result (14).

Cochran’s Q statistic was calculated to assess heterogeneity in our Mendelian Randomization analysis, with a p-value less than 0.05 indicating the presence of heterogeneity. To analyze the robustness of our results and identify potential horizontal pleiotropy, sensitivity analyses were conducted, employing MR-Egger intercept test, MR-PRESSO test, and the leave-one-out approach. The MR-Egger regression intercept was used to indicate directional pleiotropy, with a p-value less than 0.05 signifying its presence. Additionally, the MR-PRESSO test was utilized to detect outliers associated with horizontal pleiotropy, while the leave-one-out method was applied to determine if the causal association was driven by any individual SNP. In this approach, each SNP associated with the exposure was sequentially removed, followed by a repeated IVW analysis.

All analyses were conducted using the open-source statistical software R (version 4.2.0). Primarily, we utilized the TwoSampleMR (version 0.5.7), MR-PRESSO (version 1.0) for all analyses. Causal estimates were presented with 95% confidence intervals (CI), and associations with a p-value < 0.05 were considered to have suggestive significance.

## Results

### Bidrectional association between PF and LC

**Figure 2:**
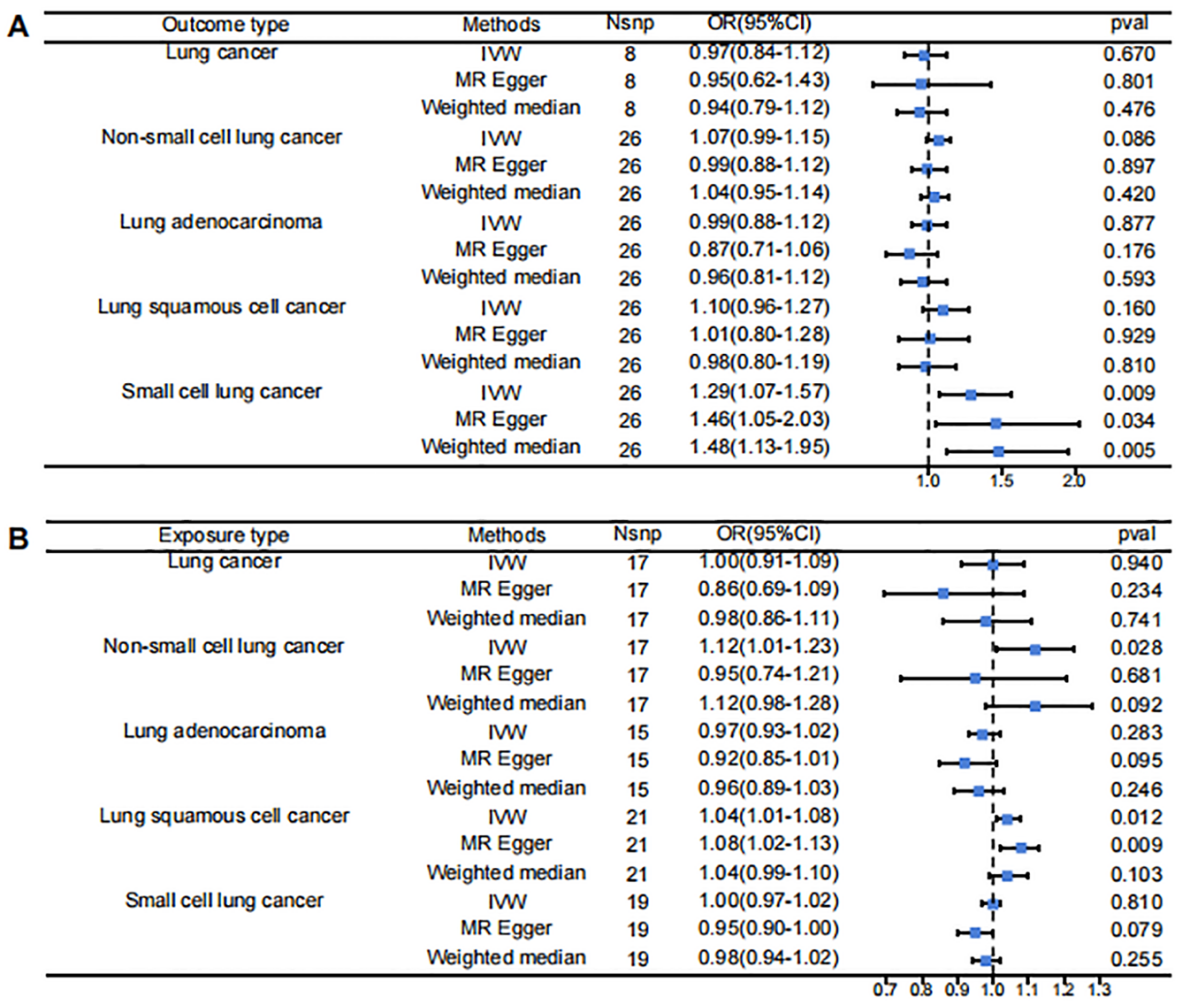
Forest Plot for Mendelian randomization results. **(A)** pulmonary fibrosis as exposure, lung cancer subtypes as outcomes **(B)** pulmonary fibrosis as exposure, lung cancer subtypes as outcomes.

The results of the bidirectional Mendelian randomization are presented in a forest plot in **Figure 2**. **Figure 2A** shows the analysis with PF as the exposure and lung cancer and its subtypes as the outcomes. **Figure 2B** illustrates the analysis with lung cancer and its subtypes as the exposures and PF as the outcome.

The IVW results indicated that when PF was the exposure, it showed a statistically significant positive association with SCLC (OR = 1.29, 95% CI 1.07-1.57, p = 0.009). However, there was no significant causal relationship between PF and LC or its other subtypes: PF and LC (OR = 0.97, 95% CI 0.84-1.12, p = 0.670), PF and NSCLC (OR = 1.07, 95% CI 0.99-1.15, p = 0.086), PF and LUAD (OR = 0.99, 95% CI 0.88-1.12, p = 0.887), PF and LUSC (OR = 1.10, 95% CI 0.96-1.27, p = 0.160).

Reverse MR analysis found that when various types of lung cancer were considered as exposures, the IVW results showed statistically significant positive associations between NSCLC and PF (OR = 1.12, 95% CI 1.01-1.23, p = 0.028) and between LUSC and PF (OR = 1.04, 95% CI 1.01-1.08, p = 0.012). However, there were no significant causal relationships between PF and LC (OR = 1.00, 95% CI 0.91-1.09, p = 0.940), LUAD (OR = 0.97, 95% CI 0.93-1.02, p = 0.283), or SCLC (OR = 1.00, 95% CI 0.97-1.02, p = 0.810).

### Results of sensitivity analysis

The MR-Egger intercept analysis indicated no evidence of horizontal pleiotropy across the MR analyses (P > 0.05). Regarding heterogeneity, even after removing SNPs with heterogeneity using MR-PRESSO, no heterogeneity remained in the MR analyses. The sensitivity analyses for the forward and reverse Mendelian randomization results are presented in **Tables 2** and **3**, respectively.

**Table 2:** Sensitivity analysis results for the analysis with PF as the exposure and lung cancer as the outcome.

	P-value			
Outcome type	Heterogeneity		Pleiotropy	MR-Presso
	IVW	MR Egger		
Lung cancer	0.997	0.991	0.905	0.264
Non-small cell lung cancer	0.148	0.194	0.160	0.099
Lung adenocarcinoma	0.177	0.247	0.120	0.878
Lung squamous cell cancer	0.376	0.369	0.368	0.172
Small cell lung cancer	0.458	0.446	0.379	0.466

**Table 3:** Sensitivity analysis results for the analysis with lung cancer as the exposure and PF as the outcome.

	P-value			
Exposure type	Heterogeneity		Pleiotropy	MR-Presso
	IVW	MR Egger		
Lung cancer	0.474	0.528	0.207	0.505
Non-small cell lung cancer	0.425	0.492	0.181	0.449
Lung adenocarcinoma	0.627	0.722	0.172	0.261
Lung squamous cell cancer	0.450	0.565	0.112	0.497
Small cell lung cancer	0.715	0.897	0.060	0.790

### Meta-analysis of the inverse-variance weighted results

**Figure 3:**
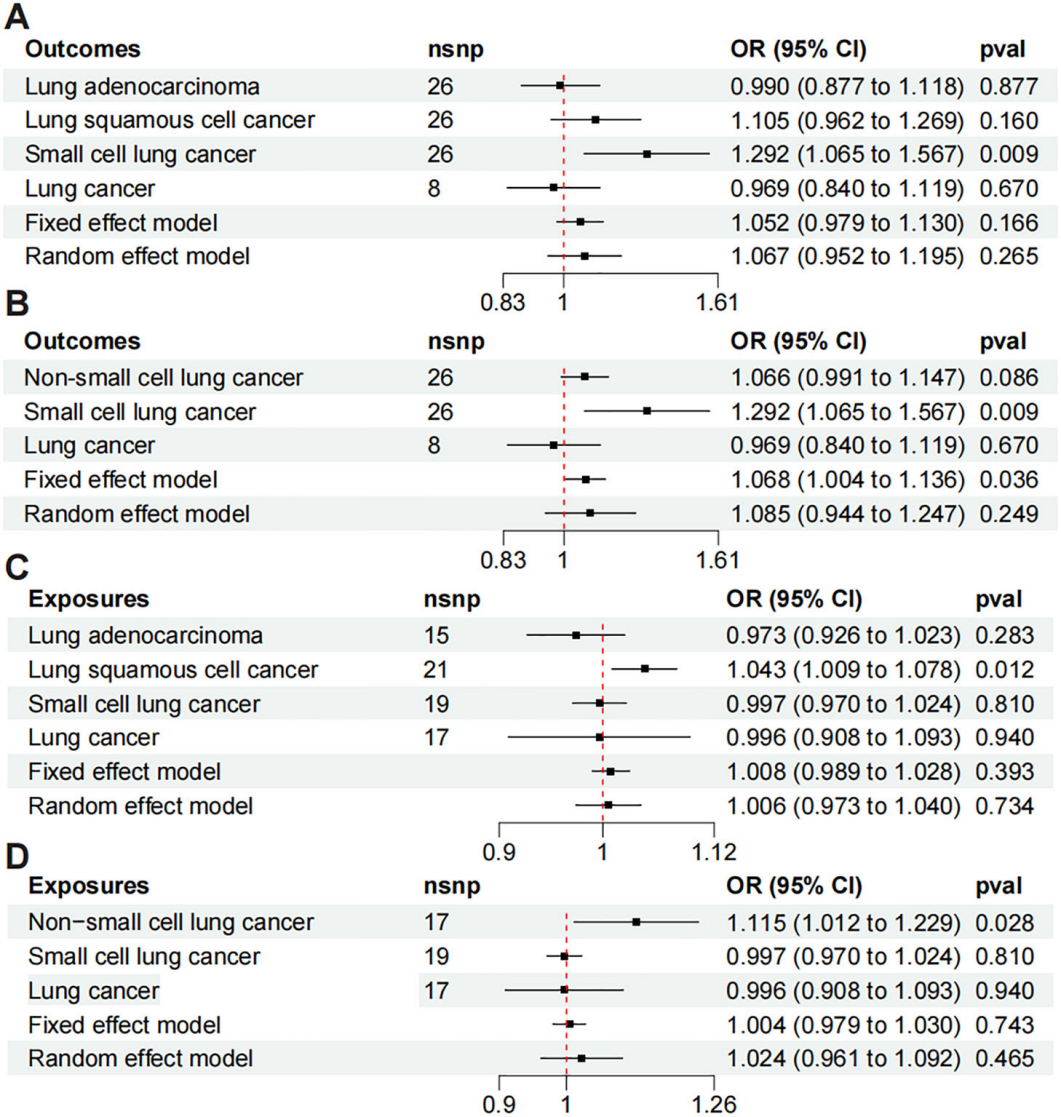
Meta-analysis of Mendelian randomization includes the following analyses: **(A)** meta-analysis of LUAD, LUSC, SCLC, and LC to PF; **(B)** meta-analysis of PF to NSCLC, SCLC, and LC; **(C)** meta-analysis of LUAD, LUSC, SCLC, and LC to PF; and **(D)** meta-analysis of NSCLC, SCLC, and LC to PF.

All the meta-analysis results exhibit heterogeneity, indicating that the random effects model is more reliable. **Figure 3A** shows the meta results for LUAD, LUSC, SCLC, and LC. When PF is the exposure, the random effects model demonstrates a non-significant positive causal relationship (OR = 1.067, 95% CI: 0.952-1.195, P = 0.265, I² = 57.3%). **Figure 3B** presents the meta results for NSCLC, SCLC, and LC. With PF as the exposure, the random effects model again shows non-significant results (OR = 1.085, 95% CI: 0.944-1.247, P = 0.249, I² = 63.6%). **Figures 3C, D** are meta-analyses with LC as the exposure and PF as the outcome. **Figure 3C** combines LUAD, LUSC, SCLC, and LC showing a result where the random effects model indicates a non-significant positive causal trend (OR = 1.006, 95% CI: 0.973-1.040, P = 0.734, I² = 55.9%). Similarly, **Figure 3D** shows meta results also non-significant (OR=1.024, 95% CI: 0.961-1.092, P = 0.465, I² = 58.1%).

## Discussion

In our study, we found a potential causal relationship between PF and SCLC, as well as between NSCLC and LUSC with PF. However, further exploration of the overall causal relationship between PF and LC did not yield statistically significant results. Compared to previous Mendelian randomization studies on IPF and LC (12), our research provides a more comprehensive investigation into the relationship between PF and LC, further elucidating the potential causal connections between these conditions.

Although SCLC has a lower incidence compared to other types of lung cancer, multiple clinical studies have demonstrated that the coexistence of SCLC and PF leads to poorer patient prognosis (15, 16). Our study indicates a potential causal relationship between PF and SCLC. Clinically, the presence of SCLC may suggest that localized pulmonary fibrosis has already developed. For such patients, it is crucial to monitor the disease progression more closely (17). LUSC is a subtype of NSCLC and is clinically considered to be highly associated with smoking. A large meta-analysis involving 7 million individuals found that male smokers have a 7.33 times higher probability of developing lung squamous cell carcinoma compared to non-smokers. For female smokers, the probability is 6.99 times higher than that of non-smokers (18). Smoking is also a risk factor for PF (19, 20). However, the specific mechanisms by which smoking contributes to the development of pulmonary fibrosis remain unclear and require further investigation (19). Therefore, we believe that smoking plays a significant role in the comorbidity of these two diseases. NSCLC, as a major subtype of lung cancer, also exhibits a causal relationship with PF, which includes both LUAD and large cell lung cancer. Research has shown that LUAD is similarly associated with smoking (21), supporting the clinical plausibility of a causal relationship between NSCLC and PF. Overall, there appears to be a positive correlation between PF and LC, though it is not statistically significant. This lack of significance may be due to individual variability in the development of PF among lung cancer patients. Additionally, the potential for PF to lead to lung cancer could be influenced by factors such as family history and other personal medical histories. In molecular mechanisms, cancer-associated fibroblasts (CAFs) are considered similar to myofibroblasts in IPF and are key components of the tumor microenvironment. In lung cancer, CAFs can originate from resident fibroblasts, cancer-associated adipocytes (CAAs), bone marrow-derived mesenchymal stromal cells (BM-MSCs), hematopoietic stem cells (HSCs), epithelial cells through epithelial-mesenchymal transition (EMT), and vascular endothelial cells through endothelial-mesenchymal transition (EndMT). Tumor cells secrete growth factors like TGF-β, EGF, and VEGF, promoting CAF transformation. CAFs increase tumor stiffness, impair vascular function, cause hypoxia, and reduce the efficacy of anticancer drugs (22). Atsushi et al.’s study analyzed DNA methylation in 20 LUSC samples and non-cancerous lung tissue samples, identifying low- and high-methylation epigenotypes. They found that low-methylation LUSC was significantly associated with IPF and had poorer prognosis, serving as an independent predictor of poor outcomes. This suggests that methylation levels may play a crucial role in the relationship between LUSC and IPF (23). In summary, there is a notable connection between PF and LC, and the coexistence of PF and LC often indicates a poor prognosis (24).

Some strengths of this study should be highlighted. As a Mendelian randomization study, it further elucidates the previously suggested potential causal relationship between PF and LC. Additionally, our study design is more comprehensive, encompassing various subtypes of lung cancer and performing a meta-analysis of all subtypes, thus providing more robust evidence of the relationship. Furthermore, we conducted reverse Mendelian randomization to explore potential reverse causality between PF and LC. However, this study also has some limitations. The original data are exclusively from European populations, which means the conclusions may only be applicable to Europeans. The selection criteria used in this study were relatively broad. Although we tested for heterogeneity, absolute rigor was not ensured. Nevertheless, previously published studies have used similar instrumental variable selection criteria (25), so we believe the conclusions are not significantly biased. Lastly, our study employed a new method to assess the causal association between PF and LC, including its subtypes. It is important to acknowledge that this method cannot distinguish between specific patient populations. It remains uncertain how the association might vary with different ages and sexes, and individual differences in this conclusion need clarification. Clinicians must use their experience and patient-specific factors to make judgments about the condition. Large-scale clinical studies are warranted, and the complex molecular mechanisms underlying the interaction between PF and LC should be further investigated.

## Data Availability

The original contributions presented in the study are included in the article/supplementary material. Further inquiries can be directed to the corresponding author.
